# Perceived Stigma towards Leprosy among Community Members Living Close to Nonsomboon Leprosy Colony in Thailand

**DOI:** 10.1371/journal.pone.0129086

**Published:** 2015-06-05

**Authors:** Nils Kaehler, Bipin Adhikar, Shristi Raut, Sujan Babu Marahatta, Robert Sedgwick Chapman

**Affiliations:** 1 Sandefjord Helsepark, Skiringssalveien 20, Sandefjord, Norway; 2 Nepal Community Health and Development Centre, Kahmandu, Nepal; 3 Department of Microbiology, Lumbini Medical College, Palpa, Nepal; 4 Department of Community Medicine, Manmohan Memorial Medical College, Kathmandu, Nepal; 5 College of Public Health Sciences, Chulalongkorn University, Bangkok, Thailand; University of Perugia, ITALY

## Abstract

**Background:**

Interpretation of Leprosy as a sickness differs among society. The set of beliefs, knowledge and perceptions towards a disease play a vital role in the construction of stigma towards a disease. The main purpose of this study was to explore the extent and correlates of the perceived stigma towards leprosy in the community living close to the leprosy colony in Non Somboon region of Khon Kaen Province of Thailand.

**Methods:**

A cross-sectional study was conducted among 257 leprosy unaffected community participants, above the age of 18 who were living close to the Leprosy colony in Non Somboon region of Thailand. Each participant was asked a questionnaire containing characteristics of the participants in terms of socio-demographic background and knowledge regarding the disease. In addition perceived stigma towards leprosy was measured using EMIC (Explanatory Model Interview Catalogue) questionnaire.

**Results:**

Among EMIC items, shame or embarrassment in the community due to leprosy was felt by 54.5%, dislike to buy food from leprosy affected persons were 49.8% and difficulty to find work for leprosy affected persons were perceived by 47.1%. Higher total EMIC score was found in participants age 61 years or older (p = 0.021), staying longer in the community (p = 0.005), attending fewer years of education (p = 0.024) and who were unemployed (p = 0.08). Similarly, perceptions about leprosy such as difficult to treat (p = 0.015), severe disease (p = 0.004) and punishment by God (p = 0.011) were significantly associated with higher perceived stigma.

**Conclusions:**

Perceived stigma towards leprosy was found highest among participants with age 61 years or older, longer duration of stay in community close to the leprosy colony, lower duration of education and participants who were unemployed had higher perceived stigma. Similarly, participants with perceptions of leprosy such as difficult to treat, severe disease and punishment by God had higher perceived stigma towards leprosy. There is an urgent need of stigma reduction strategies focused on education and awareness concerning leprosy.

## Introduction

Stigma in leprosy is a social process of interpretation which follows labeling, stereotyping, separation, resultant discrimination and the loss of status [1, 2]. In the event of leprosy, the disease is perceived by the health workers according to existing physical symptoms; the illness is experienced and shaped by the socio-cultural influences of the person; and the sickness is perceived by the society which is expressed as social stigma [3]. For any disease, an attribute alone is not creditable for the stigma attached to it but social interpretation of an attribute which is often stereotyped [4]. The rationale that motivates stigma for different conditions varies between a mix of cultural meaning, avoidance of socially discomforting disfigurement and disability, and exaggerated fear of danger and contagion. However, for leprosy the cultural meaning of the disease is an especially important feature of stigma [5]. In Thailand, leprosy is believed to be incurable and hereditary because the community often saw many cases of leprosy in one family. Villagers were thus proscribed from allowing their children to marry people with leprosy. Similarly, a man with leprosy was not allowed to enter the monkhood, a position regarded with high respect by Thai people [6]. Begging was often the obliged work for leprosy affected person which is considered as the most disgraced occupation in Thailand [7]. In Thai culture, “leprosy” and “leprosy with disability” are still translated as *Khi ruan* and *Khi thut* to degrade another person [6, 8]. Therefore, measurement of perceived stigma towards leprosy in community members is a significant means of reflecting the attitudes and the stereotypes attached to leprosy in a particular society [9].

Considering the severity in terms of human suffering, the consequences of stigma in leprosy often outweigh the burden of physical afflictions [10]. Leprosy and its stigma have a pervading effect on a patient’s life, affecting marriage, interpersonal relationships, employment, leisure activities as well as attendance at religious and social functions [11] while the extent and types of stigma can disproportionately vary between the different cultures and countries [12]. Stigma also affects the psychosocial well-being of the affected person where social consequences of leprosy can have devastating effect on their families too [13]. A person may feel fear or shame which can lead to anxiety and depression [14]. In a study conducted in Nepal, the most common reason for concealment, low self-esteem and shame or embarrassment felt by leprosy affected persons was found to be the fear of discrimination, rejection and isolation from the society [15].

Leprosy has been a social disease because of its recognition throughout the history and the attached stereotypes with it. Stigma attached with leprosy is a result of social perceptions and therefore an exploration of knowledge, attitudes and perceptions of society towards leprosy is an important reflector of stigma attached to it. In a study conducted in Indonesia, unemployment in community members was found associated with higher perceived stigma towards leprosy [16]. Similarly, in India [17] stigma towards leprosy was found higher in older patients and was associated with community subjects with lower education and lower socio-economic class. In eastern Nepal [18], stigma towards leprosy was found associated with fear of infection by germs, fear of curse by God and the deformity caused by leprosy. Similarly, in a study of perceived stigma in community members living close to Leprosy treatment center in western Nepal, perceptions such as “leprosy is difficult to treat”, and “is a severe disease” were found associated with higher level of perceived stigma [9]. In Myanmar, the lack of knowledge regarding leprosy and perceptions were attributed to the stigma addressing the urgent need of health education [19].

In Thailand, leprosy is still a stigmatizing condition. Leprosy-affected people are still stigmatized by health providers and by their neighbors [20]. Some leprosy patients have been shunned and refused treatment of their ulcers by nurse aids, resulting in delay in diagnosis and poor compliance to treatment [6, 21]. In a study involving leprosy affected persons conducted in Non-Somboon leprosy colony, higher perceived stigma was associated with some perceptions (leprosy is difficult to treat, leprosy is highly infectious and leprosy is a severe disease), ulcers, disabilities and the resultant loss of occupation due to leprosy [22] which was consistent with a study conducted in Nepal [23]. However, a study concerning a community attitudes towards leprosy in Thailand has rarely been done in past. As stereotypes prevalent in a society is not only a significant component to shape up the stigma but is also a major element that reflects the disease interpretation in a society. Therefore, we hypothesized that there is association between the levels of perceived stigma in leprosy unaffected community members and the factors characterizing them (socio-demographic characteristics, knowledge and perceptions about leprosy). While rare researches have been done in Thailand concerning stigma towards leprosy, few of them only have been published. There have been so far, no researches in particular, about the perceived stigma and in the community living close to Non somboon leprosy colony. The specific objective of this study was to determine the prevalence of perceived stigma in community members living close to Non Somboon leprosy colony and its association with factors such as socio-demographic characteristics, knowledge and the perceptions regarding leprosy.

## Methods

The design of the study was cross-sectional (S1 File). The study population comprised of community people neighboring the Non Somboon leprosy colony in Khon Kaen province, Thailand. Non Somboon leprosy colony had approximately 600 leprosy affected persons during our study period. All leprosy affected persons had completed multi-drug treatment for leprosy [22]. The community surrounding the leprosy colony was selected in order to assess the attitudes of these people towards leprosy affected persons and the colony while they live in the same community. The community stigma in this particular context can provide the clearer picture of stigma, level of acceptance in the society and need of stigma reduction programs. The community settlement was around the colony approximately at the radius of 4km. The community had 750 households in total. Two hundred and fifty seven community people were interviewed. Community subjects were selected by two stage sampling method. In the first stage, purposive selection of the community within 4 km radius of the colony was done. In the second stage, the sample frame was drawn from the community authority office and simple random sampling was applied to select the households. Consequently, one participant per household was asked for the consent before participation. Each participant was approached at their households and face to face interview was conducted. Any person from the household could participate regardless of gender but age above 18 and only those unaffected by leprosy at present and at past. A total of approximately, 20 people from the community specifically denied to participate either due to lack of time or lack of interest, however, another member of the household agreed to participate thereby resulting in a total sample size of 257 persons. Among 5 households, where interviewers could not find people were sought the consecutive days, and included in the study. None of the households during the study period was found abandoned permanently. The study was conducted from January 2013 to April 2013 after the ethical permission was obtained from Chulalongkorn University Ethical Committee.

A questionnaire (S2 File) was developed to assess socio-demographic characteristics (age, sex, ethnicity, marital status, type of family, socio-economic conditions, knowledge about leprosy and the perceptions concerning leprosy). All questions in the questionnaire form were translated from English into Thai language with the consultation of 3 experts including the linguistic and were back-translated into English for the validity. Among three experts, one of them was a social scientist, linguistics and native Thai speaker. The face-to-face interviews were performed by study-investigators who were trained prior to the start of the study. Three days training was conducted at the study site with 10 paid health workers provided by Ministry of Public Health who were doing internships at local health care centers. Training was conducted to comprehend the investigators with the introduction of the study, methodology of questionnaire administration and need of consultation. Any confusion or problem encountered was dealt by the principal investigator. Pilot testing of the questionnaire was conducted on 15 leprosy unaffected subjects. Minor corrections were done in the questionnaire to avoid few ambiguities applying translation and back translation with the consultation of linguistics.

In addition, the Explanatory Model Interview Catalogue (EMIC) scale questionnaire was used in each participant to assess the level of perceived stigma in leprosy. The EMIC scale has been developed to elicit illness-related perceptions, beliefs and the practices [24]. The EMIC questionnaire has 15 items related to perception of stigma towards leprosy. Each question is scored as “Yes = 2, Possibly = 1, No and Don’t know = 0”. EMIC scale has been both validated and reliable as evident from study in India [25] and Indonesia [26]. Descriptive statistics such as frequency, percentage, mean, median and standard deviation were used to describe the socio-economic characters and knowledge level of the participants. Difference in total perceived stigma score using EMIC between different categorical variables were analyzed using Mann Whitney U test and Kruskal Wallis H test since these scores were not normally distributed.

### Ethics Statement

The study was approved by the ethical committee of the College of Public Health Sciences at Chulalongkorn University. The approval certificate for study title “Risk factors of perceived stigma in leprosy affected and non-affected persons in Non Somboon, Khon Kaen Province, Thailand” was given on 20^th^ March 2013. All study participants received a full explanation about the study, its objectives and its benefits. All study participants could withdraw from the study at any time and without giving any reason. Informed signed consent was obtained from each participant before the onset of interview. Interview was conducted carefully considering willingness to participate and freedom of withdrawal. Anonymity of the subjects in the study was secured by coding each participant’s questionnaire form.

## Results

### Socio-demographic Characteristics

Among 257 community participants interviewed, median score of EMIC for perceived stigma was higher among the age group 61 years or above (p = 0.021). Similarly, participants who stayed in the community for longer duration had higher EMIC score (p = 0.005). Years of education had inverse relationship with EMIC score; those who attended fewer years of education had higher EMIC score (p = 0.024). Among different occupation groups, unemployed had higher EMIC score compared to farmer, laborer, private business and other (p = 0.008) (Table 1).

**Table 1:** Socio-demographic characteristics of unaffected participants and EMIC score (n = 257).

Characteristics	Number (%)	(EMIC)Median	P-value
**Age Groups (n = 257)**			
60 years or below	178(69.3)	14	**0.021** *
61 years or older	79(30.7)	18	
SD = 17.161, Median = 52, Range = 19–96			
**Sex (n = 257)**			
Female	183(71.2)	16	0.748
Male	74(28.8)	15	
**Marital status (n = 257)**			
Relationship	222(86.4)	16	0.288
No relationship	35(13.6)	14	
**Years living in community (n = 257)**			
≤ 20 years	33(12.8)	10	**0.005** *
21–40 years	63(24.5)	14	
41–60 years	100(38.9)	16.5	
≥ 61 years	54(21)	18	
**Education (n = 257)**			
Literate	251(97.7)	16	0.841
Illiterate	6(2.3)	16.5	
**Years of education (n = 251)**			
Primary level (≤ 4years)	171(68.1)	17	**0.024** **
Secondary level (5–9years)	53(21.1)	16.5	
Tertiary level (≥10years)	27(10.8)	16	
**Occupation (n = 257)**			
Farmer	75(29.2)	17	**0.008** **
Laborer	57(22.2)	13	
Private Business	38(14.8)	13	
Unemployed	44(17.1)	20	
Other	43(16.7)	16	
**Enough income to support family (n = 257)**			
Yes	120(46.7)	15.5	0.874
No	136(52.9)	16	
**Affected person in family (n = 257)**			
Yes	58(22.7)	16	0.846
No	194(75.8)	15	
Don't know	4(1.6)	18.5	
**Affected person among relative (n = 257)**			
Yes	30(11.7)	14.5	0.598
No	175(68.4)	16	
Don't know	51(19.9)	16	

*Man Whitney U test,

**Kruskal Wallis H test

### EMIC Profile

**Fig 1:**
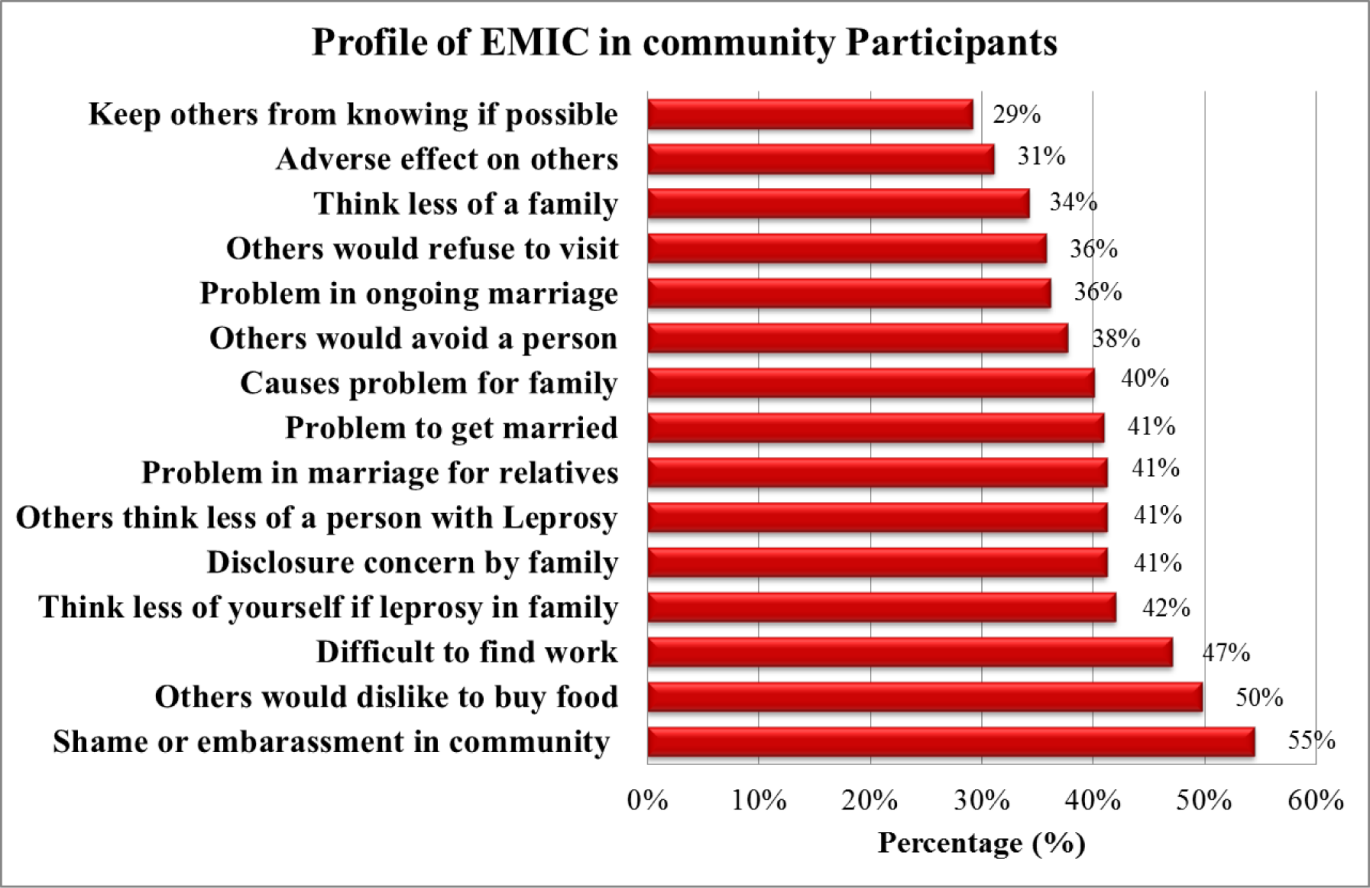
EMIC items of community participants answering “Yes” in percentage.

Explanatory Model Interview Catalogue (EMIC) score was assessed for the measurement of perceived stigma in community participants. The total median score of EMIC scale was analyzed to compare between different groups. Each domain of EMIC scale has been shown in Fig 1 with the percentage answering “yes”. More than half of the participants (54.5%) perceived shame or embarrassment in community due to leprosy. Similarly, dislike to buy foods from leprosy affected persons was perceived by 49.8% and difficult to find work for leprosy affected person was perceived by 47.1%.

### Knowledge about leprosy and perceived stigma

Almost half (47.9%) of the participants received information on leprosy. More than knowledge itself, participants’ perceptions regarding leprosy were found highly associated with higher EMIC score. Those who perceived leprosy as difficult to treat had higher EMIC score (p = 0.015). Similarly, those who perceived leprosy as severe disease (p = 0.004) and leprosy as a result of punishment by God (p = 0.011) had higher EMIC score (Table 2).

**Table 2:** Knowledge on leprosy of the participants and EMIC score (n = 257).

Characteristics	Number (%)	(EMIC)Median	P-value
**Received information on leprosy (n = 257)**			
Yes	123 (47.9)	15	0.98
No	134 (52.1)	16	
**Source of information (n = 123)**			
Medical institution	47 (38.2)	16	0.654
Friend or family	23 (18.7)	14	
Other (TV/Radio/Paper)	53 (43.1)	16	
**Knowledge on cause of leprosy (n = 257)**			
Yes	79 (30.7)	15	0.896
No	178 (69.3)	16	
**Source of leprosy cause (n = 79)**			
Bacteria/Microorganism	20 (25.3)	14.5	0.148
Other	59 (74.7)	15	
**Leprosy very infectious (n = 253)**			
Yes	105 (41.5)	17	0.054
No	148 (58.5)	14	
**Leprosy transmission (n = 257)**			
Yes	83 (32.3)	16	0.439
No	174 (67.7)	15	
**Leprosy transmitted from (n = 83)**			
Air	12 (14.5)	15.5	0.766
Water/soil	1 (1.2)	19	
Food	1 (1.2)	21	
Close contact to persons	32 (38.6)	16.5	
Other (Animal/Mosquito)	37 (44.6)	16	
**Leprosy difficult to treat (n = 257)**			
Yes	136 (52.9)	17	0.015*
No	121 (47.1)	14	
**Knowledge on signs/symptoms of leprosy**			
Yes	106 (41.2)	16	0.929
No	151 (58.8)	15	
**Signs and symptoms (n = 106)**			
Patches	21 (19.8)	14	0.861
Patches with decreased sensitivity	10 (9.4)	14	
Weakness had, feet, eyelids	1 (0.9)	15	
Nerve pain	1 (0.9)	21	
Painless wounds	3 (2.8)	16	
Multiple	70 (66.0)	17	
**Leprosy severe disease (n = 257)**			
Yes	138 (53.7)	17.5	0.004*
No	119 (46.3)	14	
**Leprosy punishment by God (n = 257)**			
Yes	72 (28.0)	18.5	0.011*
No	185 (72.0)	14	

*Man Whitney U test,

**Kruskal Wallis H test

## Discussion

Measurement of perceived stigma towards leprosy affected persons is a significant means of reflecting the attitudes and the stereotypes attached to leprosy in a society. In this study, perceived stigma was measured with EMIC scale in community people towards leprosy. Among the EMIC items (Fig 1), the major aspect affected was the shame or embarrassment in community due to presence of leprosy (54.5%). This has been well reflected by the quantitative analysis in the same study (Table 1) which showed that people living in the community for longer duration had higher level of perceived stigma. Higher level of stigma in those people who lived close to leprosy colony for longer duration is perhaps because of lack of acceptance and lack of effective community integration. However, this has been inconsistent with a study conducted in Nepal where longer duration of stay close to the leprosy hospital showed lower level of stigma [9]. This might have been because of integration of leprosy affected persons in the community. In congruence with the impact of integration on stigma, lack of integration and effective participation of leprosy affected persons in leprosy treatment program has shown higher level of stigma in India [27]. This has a major implication to scientists, health care workers and the policy makers. This warrants the urgent need of programs to increase the social participation of leprosy affected persons, integration into society and increase awareness regarding the disease which eventually might reduce the level of non-acceptance and perceived stigma towards leprosy affected persons.

Dislike to buy food from leprosy affected persons was perceived by 49.8% which was consistent with studies conducted in Indonesia [16] and Nepal [9]. This might have been because of the fear of infection, beliefs and the perceptions about leprosy [11]. Difficult to find work for leprosy affected person was perceived by 47.1%. This has shown relative consistencies with the study done in leprosy affected persons within Nonsomboon leprosy colony where higher perceived stigma was found in those subjects who were obliged to leave their occupation due to leprosy [22]. Similar results were found in studies done in Nepal [23, 28]. Marriage problems for the person affected by leprosy and their family members were perceived to be significantly affected by the disease condition which has been consistent with a study conducted in Indonesia [16].

Older age (≥61 years) was found associated with higher level of perceived stigma in community participants (p = 0.021). The association of older age with higher level of stigma in this study might show the level of unchanged stereotypical views towards leprosy. This has a significant implication, which might demonstrate the level of non-acceptance and lack of social integration over the years. Similar results were found in studies conducted in India where stigma was higher in subjects above the age of 46 years [17, 29].

Longer duration of stay in the community close to leprosy colony had higher perceived stigma. People who lived in the community longer had progressively higher stigma compared to those who lived fewer years (p = 0.005). This shows consistencies with our own results, where older people had higher level of stigma and more than 50% of subjects perceived that they would feel shame or embarrassment in community due to presence of leprosy. This might have been because of persistence of stereotypical views towards leprosy in older subjects. In addition, this might reflect the lack of stigma reduction strategies such as health education, social integration and integrated health approach. The evidence of integrated health approach in reducing stigma is shown by a study in India where 50% reduction in level of stigma was found with community participation in leprosy control program [27]. Furthermore, a study in Brazil shows the main challenges in leprosy control program as lack of leprosy care at local health services and social mobilization of the community to leprosy control program [30].

Lower level of education was associated with higher level of perceived stigma (p = 0.024). Level of education and level of perceived stigma have been found to have the linear relationship in a study conducted in Nepal [23] and India [17]. The impact of education on perceived stigma score could be to increase the overall knowledge on disease and an increased ability to resist the negative stereotype attached with the disease [23].

Unemployment in community has been found associated with higher level of perceived stigma. This has been consistent with the Indonesian study where unemployment in community members was found associated with higher level of perceived stigma [16]. Unemployment and farming are likely to be associated with lower socio-economic condition and lower level of education which might be responsible for higher level of perceived stigma. The lower socio-economic condition and lower level of education have been found to be associated with higher perceived stigma in many other studies [17, 31–34].

Perceptions such as leprosy is difficult to treat, severe disease and a result of punishment by God have been found to have higher perceived stigma. This has been consistent with a study conducted with community members [9] and leprosy affected persons in Western Nepal [23] and in Thailand [22]. Fear of infection [18, 35], and beliefs [35–37] such as curse by God [18] were found to be major factors associated with stigma in Nepal [18, 36], India [33] China [35], and Nigeria [37]. This shows that different types of perceptions and beliefs concerning leprosy are globally prevalent and have been the major contributors of stigma in leprosy. This has major implications to counteract the stigma by directing the stigma reduction strategies.

## Limitation

This study included the community participants staying close to Non Somboon leprosy colony in Khon Kaen province therefore cannot be generalized for the population living far from the colony and rest of the regions of Thailand. This study focused on the perceived stigma in community participants while rest of the types of stigma such as self-stigma and enacted stigma were not accounted therefore stigma from this study cannot be generalized for all types of stigma. This study did not include the multiple regression analysis which limits the strength of the study.

## Conclusion

This study concludes the significant amount of perceived shame or embarrassment due to the presence of leprosy colony in the community in addition to some other aspects such as refusal to buy food from leprosy affected persons and perceived difficulty to find work for a person affected by leprosy. The higher level of stigma in older age group and those living longer in the community also reflects the level of stigma present in the community and the level of acceptance. This study further concludes that the higher level of stigma is associated with lower level of education, unemployment and perceptions such as difficult to treat, severe disease and punishment by God which warrants the need of interventional programs to counteract the stigma. Stigma reduction strategies, such as health education, community participation and social integration might reduce the level of stigma and consequently can increase the social acceptance.

## Supporting Information

S1 File STROBE Checklist for cross-sectional studies. (DOC)

S2 File Leprosy research questionnaire for community members. (DOCX)
