# Correction: Perceived Stigma towards Leprosy among Community Members Living Close to Nonsomboon Leprosy Colony in Thailand

**DOI:** 10.1371/journal.pone.0135537

**Published:** 2015-08-06

**Authors:** The PLOS ONE Staff

The second author’s name is spelled incorrectly. The correct name is Bipin Adhikari. The correct citation is: Kaehler N, Adhikari B, Raut S, Marahatta SB, Chapman RS (2015) Perceived Stigma towards Leprosy among Community Members Living Close to Nonsomboon Leprosy Colony in Thailand. PLoS ONE 10(6): e0129086. The publisher apologizes for the error.

There are spelling errors in Tables 1 and 2. Please see the correct Tables 1 and 2, provided by the authors, here.

**Table 1:** Socio-demographic characteristics of unaffected participants and EMIC score (n = 257)

Characteristics	Number (%)	(EMIC)Median	P-value
**Age Groups (n = 257)**			
60 years or below	178(69.3)	14	**0.021** *
61 years or older	79(30.7)	18	
SD = 17.161, Median = 52, Range = 19–96			
**Sex (n = 257)**			
Female	183(71.2)	16	0.748
Male	74(28.8)	15	
**Marital status (n = 257)**			
Relationship	222(86.4)	16	0.288
No relationship	35(13.6)	14	
**Years living in community (n = 257)**			
≤ 20 years	33(12.8)	10	**0.005** *
21–40 years	63(24.5)	14	
41–60 years	100(38.9)	16.5	
≥ 61 years	54(21)	18	
**Education (n = 257)**			
Literate	251(97.7)	16	0.841
Illiterate	6(2.3)	16.5	
**Years of education (n = 251)**			
Primary level (≤ 4years)	171(68.1)	17	**0.024** **
Secondary level (5-9years)	53(21.1)	16.5	
Tertiary level (≥10years)	27(10.8)	16	
**Occupation (n = 257)**			
Farmer	75(29.2)	17	**0.008** **
Laborer	57(22.2)	13	
Private Business	38(14.8)	13	
Unemployed	44(17.1)	20	
Other	43(16.7)	16	
**Enough income to support family (n = 257)**			
Yes	120(46.7)	15.5	0.874
No	136(52.9)	16	
**Affected person in family (n = 257)**			
Yes	58(22.7)	16	0.846
No	194(75.8)	15	
Don't know	4(1.6)	18.5	
**Affected person among relative (n = 257)**			
Yes	30(11.7)	14.5	0.598
No	175(68.4)	16	
Don't know	51(19.9)	16	

*Man Whitney U test,

**Kruskal Wallis H test

**Table 2:** Knowledge on leprosy of the participants and EMIC score (n = 257).

Characteristics	Number (%)	(EMIC)Median	P-value
**Received information on leprosy (n = 257)**			
Yes	123 (47.9)	15	0.98
No	134 (52.1)	16	
**Source of information (n = 123)**			
Medical institution	47 (38.2)	16	0.654
Friend or family	23 (18.7)	14	
Other (TV/Radio/Paper)	53 (43.1)	16	
**Knowledge on cause of leprosy (n = 257)**			
Yes	79 (30.7)	15	0.896
No	178 (69.3)	16	
**Source of leprosy cause (n = 79)**			
Bacteria/Microorganism	20 (25.3)	14.5	0.148
Other	59 (74.7)	15	
**Leprosy very infectious (n = 253)**			
Yes	105 (41.5)	17	0.054
No	148 (58.5)	14	
**Leprosy transmission (n = 257)**			
Yes	83 (32.3)	16	0.439
No	174 (67.7)	15	
**Leprosy transmitted from (n = 83)**			
Air	12 (14.5)	15.5	0.766
Water/soil	1 (1.2)	19	
Food	1 (1.2)	21	
Close contact to persons	32 (38.6)	16.5	
Other (Animal/Mosquito)	37 (44.6)	16	
**Leprosy difficult to treat (n = 257)**			
Yes	136 (52.9)	17	**0.015** *
No	121 (47.1)	14	
**Knowledge on signs/symptoms of leprosy**			
Yes	106 (41.2)	16	0.929
No	151 (58.8)	15	
**Signs and symptoms (n = 106)**			
Patches	21 (19.8)	14	0.861
Patches with decreased sensitivity	10 (9.4)	14	
Weakness hand, feet, eyelids	1 (0.9)	15	
Nerve pain	1 (0.9)	21	
Painless wounds	3 (2.8)	16	
Multiple	70 (66.0)	17	
**Leprosy severe disease (n = 257)**			
Yes	138 (53.7)	17.5	**0.004** *
No	119 (46.3)	14	
**Leprosy punishment by God (n = 257)**			
Yes	72 (28.0)	18.5	**0.011** *
No	185 (72.0)	14	

*Man Whitney U test,

**Kruskal Wallis H test
