# Supplementary material for: Perceived Stigma towards Leprosy among Community Members Living Close to Nonsomboon Leprosy Colony in Thailand
Source: PLoS One. 2015 Jun 5;10(6):e0129086. doi: 10.1371/journal.pone.0129086 (PMC4457619; doi:10.1371/journal.pone.0129086)
Supplement: S1 File — (DOC) [file pone.0129086.s001.doc]

CHECKLIST S1: STROBE Checklist for cross-sectional studies

|  | Item No | Recommendation |
| --- | --- | --- |
| **Title and abstract** | 1 | (*a*) Indicate the study’s design with a commonly used term in the title or the abstract  ***Methods:*** *A cross-sectional study was conducted among 257 leprosy unaffected community participants, above the age of 18 who were living close to the Leprosy colony in Non Sombon region of Thailand. Each participant was asked with a set of questionnaire containing characteristics of the participants in terms of socio-demographic background and knowledge regarding the disease. In addition perceived stigma towards leprosy was measured using EMIC (Explanatory Model Interview Catalogue) questionnaire.* |
|  |  | (*b*) Provide in the abstract an informative and balanced summary of what was done and what was found  ***Methods:*** *A cross-sectional study was conducted among 257 leprosy unaffected community participants, above the age of 18 who were living close to the Leprosy colony in Non Sombon region of Thailand. Each participant was asked with a set of questionnaire containing characteristics of the participants in terms of socio-demographic background and knowledge regarding the disease. In addition perceived stigma towards leprosy was measured using EMIC (Explanatory Model Interview Catalogue) questionnaire).*  ***Results:*** *Among EMIC items, shame or embarrassment in community due to leprosy was felt by 54.5%, dislike to buy food from leprosy affected persons were 49.8% and difficulty to find work for leprosy affected persons were perceived by 47.1%. Higher total EMIC score was found in participants age 61 years or older (p=0.021), staying longer in the community (p=0.005), attending fewer years of education (p=0.024) and who were unemployed (p=0.08). Similarly, perceptions about leprosy such as difficult to treat (p=0.015), severe disease (p=0.004) and punishment by god (p=0.011) were significantly associated with higher perceived stigma.* |
| Introduction | | |
| Background/rationale | 2 | Explain the scientific background and rationale for the investigation being reported  ***Introduction***  *Stigma is a social process of interpretation of any disease which follows labeling, stereotyping, separation, resultant discrimination and the loss of status (1, 2). In the event of leprosy, the disease is perceived by the health workers according to existing physical symptoms; the illness is experienced and shaped by the socio-cultural influences of the person; and the sickness is perceived by the society which is expressed as social stigma (3). For any disease, an attribute alone is not creditable for the stigma attached to it but social interpretation of an attribute which is often stereotyped (4). The rationale that motivates stigma for different conditions varies between a mix of cultural meaning, avoidance of socially discomforting disfigurement and disability, and exaggerated fear of danger and contagion. However, for leprosy the cultural meaning of the disease is an especially important feature of stigma (5). In Thailand, leprosy is believed to be incurable and hereditary because the community often saw many cases of leprosy in one family. Villagers were thus proscribed from allowing their children to marry people with leprosy. Similarly, A man with leprosy was not allowed to enter the monkhood, a position regarded with high respect by Thai people (6). Begging was often the obliged work for leprosy affected person which is considered as the most disgraced occupation in Thailand (7). In Thai culture, “leprosy” and “leprosy with disability” are still translated as Khi ruan and Khi thut to degrade another person (6,8). Therefore, measurement of perceived stigma towards leprosy in community members is a significant means of reflecting the attitudes and the stereotypes attached to leprosy in a particular society (9).*  *Considering the severity in terms of human suffering, the consequences of stigma in leprosy often outweigh the burden of physical afflictions (10). Leprosy and its stigma have a pervading effect on a patient’s life, affecting marriage, interpersonal relationships, employment, leisure activities as well as attendance at religious and social functions (11) while the extent and types of stigma can disproportionately vary between the different cultures and countries (12). Stigma also affects the psychosocial well-being of the affected person where social consequences of leprosy can have devastating effect on their families too (13). A person may feel fear or shame which can lead to anxiety and depression (14). In a study conducted in Nepal, the most common reason for concealment, low self-esteem and shame or embarrassment felt by leprosy affected persons was found to be the fear of discrimination, rejection and isolation from the society (15).*  *Leprosy has been the social disease because of its adequate social recognition and social interpretation. Stigma attached with leprosy is a result of social perceptions and therefore an exploration of characteristics of society in relation to leprosy is an important reflector of stigma attached to it. In a study conducted in Indonesia, unemployment in community members was found associated with higher perceived stigma towards leprosy (16). Similarly, in India (17) stigma towards leprosy was found higher in older patients and was associated with community subjects with lower education and lower socio-economic class. In eastern Nepal (18), stigma towards leprosy was found associated with fear of infection by germs, fear of curse by god and the deformity caused by leprosy. Similarly, in a study of perceived stigma in community members living close to Leprosy treatment center in western Nepal, perceptions such as “leprosy is difficult to treat”, and “is a severe disease” were found associated with higher level of perceived stigma (9). In Myanmar, the lack of knowledge regarding leprosy and perceptions were attributed to the stigma addressing the urgent need of health education (19).*  *In Thailand, leprosy is still a stigmatizing condition. Leprosy-affected people are still stigmatized by health providers and by their neighbors (20). Some leprosy patients have been shunned and refused treatment of their ulcers by nurse aids, resulting in delay in diagnosis and poor compliance to treatment (6, 21). In a study involving leprosy affected persons conducted in Non-Somboon leprosy colony, higher perceived stigma was associated with the perceptions (leprosy is difficult to treat, leprosy is highly infectious and leprosy is a severe disease), ulcers, disabilities and the resultant loss of occupation due to leprosy (22) which was consistent with a study conducted in Nepal (23). However, a study concerning a community attitudes towards leprosy in Thailand has rarely been done in past. As stereotypes prevalent in a society is not only a significant component to shape up the stigma but is also a major element that reflects the disease interpretation in a society. Therefore, we hypothesized that there is association between the levels of perceived stigma in leprosy unaffected community members and the factors characterizing them (socio-demographic characteristics, Knowledge and perceptions about leprosy). While rare researches have been done in Thailand concerning stigma towards leprosy, few of them only have been published. There have been so far, no researches in particular, the perceived stigma and in the community living close to Non somboon leprosy colony. The specific objective of this study was to determine the prevalence of perceived stigma in community members living close to Non Somboon leprosy colony and its association with factors such as socio-demographic characteristics, knowledge and the perceptions regarding leprosy.* |
| Objectives | 3 | State specific objectives, including any pre-specified hypotheses  *The specific objective of this study was to determine the prevalence of perceived stigma in community members living close to Non Somboon leprosy colony and its association with factors such as socio-demographic characteristics, knowledge and the perceptions regarding leprosy.*  *We hypothesized that there is association between the levels of perceived stigma in leprosy unaffected community members and the factors characterizing them (socio-demographic characteristics, Knowledge and perceptions about leprosy).* |
| Methods | | |
| Study design | 4 | Present key elements of study design early in the paper  ***Materials and Methods:*** *The study was a cross-sectional in design. The study population comprised community people neighboring the Non Somboon leprosy colony in Khon Kaen province, Thailand.* |
| Setting | 5 | Describe the setting, locations, and relevant dates, including periods of recruitment, exposure, follow-up, and data collection  ***Materials and Methods:*** *The study population comprised community people neighboring the Non Somboon leprosy colony in Khon Kaen province, Thailand. Community close to leprosy colony was selected in order to assess the attitudes of these people towards leprosy affected persons and the colony while they live in the same community. The community stigma in this particular context can provide the clearer picture of stigma, level of acceptance in the society and need of stigma reduction programs. The community settlement was around the colony approximately at the radius of 4km. The community had total 750 households. Two hundred and fifty seven community people were interviewed. Community subjects were selected by two stage sampling method. In the first stage, purposive selection of the community within 4 km radius of the colony was done. In the second stage, the sample frame was drawn from the community authority office and simple random sampling was applied to select the households. Consequently, one participant per each household selected was asked for the consent before participation. Each participant was approached at their households and face to face interview was conducted. Any person from the household could participate allowing one person from one household. Each Participant was chosen regardless of gender but age above 18 and only those unaffected by leprosy at present and at past. Around 20 people from the community in total specifically denied to participate either due to lack of time or lack of interest, however, another member of the household agreed to participate thereby meeting the total sample size of 257 persons. Among 5 households, where interviewers could not find people were sought the consecutive days, and included in the study. None of the household during study period was found abandoned permanently. The study was conducted from January 2013 to April 2013 after the ethical permission was obtained from Chulalongkorn University Ethical Committee.*  *A questionnaire was developed to assess the socio-demographic characteristics (age, sex, ethnicity, marital status, type of family, socio-economic conditions, knowledge about leprosy and the perceptions concerning leprosy). All questions in the questionnaire form were translated from English into Thai language with the consultation of 3 experts including the linguistic and were back-translated into English for the validity. Among three experts, one of them was a social scientist, linguistics and native Thai speaker. The face-to-face interviews were performed by study-investigators who were trained prior to the start of the study. Three days training was conducted at study site with 10 paid health workers provided by Ministry of Public Health who were doing internships at local health care centers. Training was conducted to comprehend the investigators with the introduction of the study, methodology of questionnaire administration and need of consultation. Any confusion or problem encountered was dealt by the principal investigator. Pilot testing of the questionnaires was conducted on 15 leprosy unaffected subjects. Minor corrections were done in the questionnaire to avoid few ambiguities applying translation and back translation with the consultation of linguistics.*  *In addition, the Explanatory Model Interview Catalogue (EMIC) scale questionnaire was used in each participant to assess the level of perceived stigma in leprosy.* |
| Participants | 6 | (*a*) Give the eligibility criteria, and the sources and methods of selection of participants  *Community subjects were selected by two stage sampling method. In the first stage, purposive selection of the community within 4 km radius of the colony was done. In the second stage, the sample frame was drawn from the community authority office and simple random sampling was applied to select the households. Consequently, one participant per each household selected was asked for the consent before participation. Each participant was approached at their households and face to face interview was conducted. Any person from the household could participate allowing one person from one household. Each Participant was chosen regardless of gender but age above 18 and only those unaffected by leprosy at present and at past. Around 20 people from the community in total specifically denied to participate either due to lack of time or lack of interest, however, another member of the household agreed to participate thereby meeting the total sample size of 257 persons. Among 5 households, where interviewers could not find people were sought the consecutive days, and included in the study. None of the household during study period was found abandoned permanently.* |
| Variables | 7 | Clearly define all outcomes, exposures, predictors, potential confounders, and effect modifiers. Give diagnostic criteria, if applicable  ***Independent Variables:*** *A questionnaire was developed to assess the socio-demographic characteristics (age, sex, ethnicity, marital status, type of family, socio-economic conditions, knowledge about leprosy and the perceptions concerning leprosy.* *Total two hundred and fifty seven community people were interviewed.* *All questions in the questionnaire form were translated from English into Thai language with the consultation of 3 experts including the linguistic and were retranslated into English for the validity. The face-to-face interviews were performed by study-investigators who were trained prior to the start of the study. Pilot testing of the questionnaires was conducted on 15 leprosy unaffected subjects.*  ***Dependent Variables:*** *In addition, the Explanatory Model Interview Catalogue (EMIC) scale questionnaire was used in each participant to assess the level of perceived stigma in leprosy. The EMIC scale has been developed to elicit illness-related perceptions, beliefs and the practices (24). The EMIC questionnaire has 15 items related to perception of stigma towards leprosy. Each question is scored as “Yes = 2, Possibly = 1, No and Don’t know = 0”.*  ***Analysis:*** *Descriptive statistics such as frequency, percentage, mean, median and standard deviation were used to describe the socio-economic characters and knowledge level of the participants. Difference in total perceived stigma score using EMIC between different categorical variables were analyzed using Mann Whitney U test and Kruskal Wallis H test since these scores were not normally distributed.* |
| Data sources/ measurement | 8* | For each variable of interest, give sources of data and details of methods of assessment (measurement). Describe comparability of assessment methods if there is more than one group  *Two hundred and fifty seven community people were interviewed. Community subjects were selected by two stage sampling method. In the first stage, purposive selection of the community within 4 km radius of the colony was done. In the second stage, the sample frame was drawn from the community authority office and simple random sampling was applied to select the community subjects. Participants were chosen regardless of gender but age above age 18 and only those unaffected by leprosy at present and at past. The study was conducted from January 2013 to April 2013 after the ethical permission was obtained from Chulalongkorn University Ethical Committee.*  *A questionnaire was developed to assess the socio-demographic characteristics (age, sex, ethnicity, marital status, type of family, socio-economic conditions, knowledge about leprosy and the perceptions concerning leprosy. All questions in the questionnaire form were translated from English into Thai language with the consultation of 3 experts including the linguistic and were retranslated into English for the validity. The face-to-face interviews were performed by study-investigators who were trained prior to the start of the study. Pilot testing of the questionnaires was conducted on 15 leprosy unaffected subjects.*  *In addition, the Explanatory Model Interview Catalogue (EMIC) scale questionnaire was used in each participant to assess the level of perceived stigma in leprosy. The EMIC scale has been developed to elicit illness-related perceptions, beliefs and the practices (24). The EMIC questionnaire has 15 items related to perception of stigma towards leprosy. Each question is scored as “Yes = 2, Possibly = 1, No and Don’t know = 0”. EMIC scale has been both validated and reliable as evident from study in India (25) and Indonesia (26). Descriptive statistics such as frequency, percentage, mean, median and standard deviation were used to describe the socio-economic characters and knowledge level of the participants. Difference in total perceived stigma score using EMIC between different categorical variables were analyzed using Mann Whitney U test and Kruskal Wallis H test since these scores were not normally distributed.* |
| Bias | 9 | Describe any efforts to address potential sources of bias  *Community subjects were selected by two stage sampling method. In the first stage, purposive selection of the community within 4 km radius of the colony was done. In the second stage, the sample frame was drawn from the community authority office and simple random sampling was applied to select the households. One participant per each selected household was chosen for the interview. Therefore, selection bias was completely averted.*  *Interview was conducted carefully considering willingness to participate and freedom of withdrawal. Anonymity of the subjects in the study was secured by coding each participant’s questionnaire form.* |
| Study size | 10 | Explain how the study size was arrived at  *Primkeaw et al. described a rate of 77% of (undefined) stigma in the leprosy unaffected community. We furthermore knew that the community had total household of 750. In view of this, a perceived prevalence of 70% in the unaffected persons was estimated. Cochran formula calculated to 323 for prevalence = 0.70. In the second, step a correction for total households was performed, in this case for 750 households in the community. The correction was (provisional SS)(total households) / (provisional SS + total households), in this case (323)(750) / 1073 = 225. To account for incomplete data, 14% were added, for a sample size of 257.* |
| Quantitative variables | 11 | Explain how quantitative variables were handled in the analyses. If applicable, describe which groupings were chosen and why  ***Independent Variables:*** *A questionnaire was developed to assess the socio-demographic characteristics (age, sex, ethnicity, marital status, type of family, socio-economic conditions, knowledge about leprosy and the perceptions concerning leprosy).*  ***Dependent Variables:*** *In addition, the Explanatory Model Interview Catalogue (EMIC) scale questionnaire was used in each participant to assess the level of perceived stigma in leprosy. The EMIC questionnaire has 15 items related to perception of stigma towards leprosy. Each question is scored as “Yes = 2, Possibly = 1, No and Don’t know = 0”.*  ***Analysis:*** *Descriptive statistics such as frequency, percentage, mean, median and standard deviation were used to describe the socio-economic characters and knowledge level of the participants. Difference in total perceived stigma score using EMIC between different categorical variables were analyzed using Mann Whitney U test and Kruskal Wallis H test since these scores were not normally distributed.* |
| Statistical methods | 12 | (*a*) Describe all statistical methods, including those used to control for confounding  *Descriptive statistics were used to describe the socio-economic, knowledge level and clinical presentation of the participants. Difference in total perceived stigma score using EMIC between different categorical variables were analyzed using Mann Whitney U test and Kruskal Wallis H test since these scores were not normally distributed.* |
|  |  | (*b*) Describe any methods used to examine subgroups and interactions |
|  |  | (*c*) Explain how missing data were addressed  *There were only 4 missing data in a particular question and it has been unmodified and presented in the study as it is.* |
|  |  | (*d*) If applicable, describe analytical methods taking account of sampling strategy  *Two hundred and fifty seven community people were interviewed. Community subjects were selected by two stage sampling method. In the first stage, purposive selection of the community within 4 km radius of the colony was done. In the second stage, the sample frame was drawn from the community authority office and simple random sampling was applied to select the community subjects per each households. Participants were chosen regardless of gender but age above age 18 and only those unaffected by leprosy at present and at past. The study was conducted from January 2013 to April 2013 after the ethical permission was obtained from Chulalongkorn University Ethical Committee.* |
|  |  | (*e*) Describe any sensitivity analyses |
| Results | | |
| Participants | 13* | (a) Report numbers of individuals at each stage of study—eg numbers potentially eligible, examined for eligibility, confirmed eligible, included in the study, completing follow-up, and analysed  *Community subjects were selected by two stage sampling method. In the first stage, purposive selection of the community within 4 km radius of the colony was done. In the second stage, the sample frame was drawn from the community authority office and simple random sampling was applied to select the households. Consequently, one participant per each household selected was asked for the consent before participation. Each participant was approached at their households and face to face interview was conducted. Any person from the household could participate allowing one person from one household. Each Participant was chosen regardless of gender but age above 18 and only those unaffected by leprosy at present and at past. Around 20 people from the community in total specifically denied to participate either due to lack of time or lack of interest, however, another member of the household agreed to participate thereby meeting the total sample size of 257 persons. Among 5 households, where interviewers could not find people were sought the consecutive days, and included in the study. None of the household during study period was found abandoned permanently.* |
|  |  | (b) Give reasons for non-participation at each stage |
|  |  | (c) Consider use of a flow diagram |
| Descriptive data | 14* | (a) Give characteristics of study participants (eg demographic, clinical, social) and information on exposures and potential confounders  *It has been detailed in* ***Result*** *section*  *Among 257 community participants interviewed, median score of EMIC for perceived stigma was higher among the age group 61 years or above (p = 0.021). Similarly, participants who stayed in the community more than 21 years had higher EMIC score (p = 0.001). Years of education had inverse relationship with EMIC score; those who attended fewer years of education had higher EMIC score (p = 0.024). Unemployed and farmer as an occupation had higher EMIC score (p = 0.008) compared to other occupation such as private/government officer and students (Table 2).* |
|  |  | (b) Indicate number of participants with missing data for each variable of interest  ***Variable:*** *Is Leprosy Infectious ? Yes/No; total missing 4.* |
| Outcome data | 15* | Report numbers of outcome events or summary measures  *Socio-demographic Characteristics*  *Among 257 community participants interviewed, median score of EMIC for perceived stigma was higher among the age group 61 years or above (p = 0.021). Similarly, participants who stayed in the community for longer duration had higher EMIC score (p = 0.005). Years of education had inverse relationship with EMIC score; those who attended fewer years of education had higher EMIC score (p = 0.024). Among different occupation groups, unemployed had higher EMIC score compared to farmer, laborer, private business and other (p = 0.008) (Table 1).*  *EMIC Profile*  *Explanatory Model Interview Catalogue (EMIC) score was assessed for the measurement of perceived stigma in community participants. The total median score of EMIC scale was analyzed to compare between different groups. Each domain of EMIC scale has been shown in Figure 1 with the percentage answering “yes”. More than half of the participants (54.5%) perceived shame or embarrassment in community due to leprosy. Similarly, dislike to buy foods from leprosy affected persons was perceived by 49.8% and difficult to find work for leprosy affected person was perceived by 47.1%.*  *Knowledge about leprosy and perceived stigma*  *Almost half (47.9%) of the participants received information on leprosy. More than knowledge itself, participants’ perceptions regarding leprosy were found highly associated with higher EMIC score. Those who perceived leprosy as difficult to treat had higher EMIC score (p = 0.015). Similarly, those who perceived leprosy as severe disease (p = 0.004) and leprosy as a result of punishment by god (p = 0.011) had higher EMIC score (Table 2).* |
| Main results | 16 | (*a*) Give unadjusted estimates and, if applicable, confounder-adjusted estimates and their precision (eg, 95% confidence interval). Make clear which confounders were adjusted for and why they were included |
|  |  | (*b*) Report category boundaries when continuous variables were categorized  *Age groups were categorized into two groups with the cut-off point at 60 years.*  *Years living in community were categorized into the cut-off by 20 years.* |
|  |  | (*c*) If relevant, consider translating estimates of relative risk into absolute risk for a meaningful time period |
| Other analyses | 17 | Report other analyses done—eg analyses of subgroups and interactions, and sensitivity analyses |
| Discussion | | |
| Key results | 18 | Summarise key results with reference to study objectives  ***The specific objective*** *of this study was to determine the prevalence of perceived stigma in community members living close to Non Somboon leprosy colony and its association with factors such as socio-demographic characteristics, knowledge and the perceptions regarding leprosy.*  ***Key results*** *Among EMIC items, shame or embarrassment in community due to leprosy was felt by 54.5%, dislike to buy food from leprosy affected persons were 49.8% and difficulty to find work for leprosy affected persons were perceived by 47.1%. Higher total EMIC score was found in participants age 61 years or older (p=0.021), staying longer in the community (p=0.005), attending fewer years of education (p=0.024) and who were unemployed (p=0.08). Similarly, perceptions about leprosy such as difficult to treat (p=0.015), severe disease (p=0.004) and punishment by god (p=0.011) were significantly associated with higher perceived stigma.* |
| Limitations | 19 | Discuss limitations of the study, taking into account sources of potential bias or imprecision. Discuss both direction and magnitude of any potential bias  ***Limitations***  *This study included the community participants staying close to Non somboon leprosy colony in khon kaen province therefore cannot be generalized for the population living far from the colony and rest of the regions of Thailand. This study focused on the perceived stigma in community participants while rest of the types of stigma such as self-stigma and enacted stigma were not accounted therefore stigma from this study cannot be generalized for all types of stigma. This study did not include the multiple regression analysis which limits the strength of the study.* |
| Interpretation | 20 | Give a cautious overall interpretation of results considering objectives, limitations, multiplicity of analyses, results from similar studies, and other relevant evidence  ***Conclusion***  *Community participants showed the shame or embarrassment in community due to presence of leprosy which was the most affected item in EMIC profile. Similarly, their perceived attitudes such as refusal of buying food from leprosy affected persons and difficulty finding a work for leprosy affected persons were found as the other major items affected in EMIC. This reflects the level of non-acceptance of the leprosy colony and the persistence of false stereotypes regarding leprosy. This has been also evident by the presence of higher level of stigma in older age group and those living longer in the community. Similarly, lower level of education and unemployment had higher perceived stigma towards leprosy. Our study, showed the perceptions about the disease to be another contributors of higher stigma in community participants. Perceptions regarding leprosy such as difficult to treat, severe disease and punishment by god might be a clear reflection of society’s interpretation of the disease. This again shows serious need of interventional programs perhaps focused with health education, community participation and integration of leprosy affected persons in society. This might reduce the level of non-acceptance at community and the false stereotypes attached with the disease.* |
| Generalisability | 21 | Discuss the generalisability (external validity) of the study results  *This study included the community participants staying close to Non somboon leprosy colony in khon kaen province therefore cannot be generalized for the population living far from the colony and rest of the regions of Thailand. This study focused on the perceived stigma in community participants while rest of the types of stigma such as self-stigma and enacted stigma were not accounted therefore stigma from this study cannot be generalized for all types of stigma. This study did not include the multiple regression analysis which limits the strength of the study.* |
| Other information | | |
| Funding | 22 | Give the source of funding and the role of the funders for the present study and, if applicable, for the original study on which the present article is based  *No funding was available for this study.* |

*Give information separately for exposed and unexposed groups.

**Note:** An Explanation and Elaboration article discusses each checklist item and gives methodological background and published examples of transparent reporting. The STROBE checklist is best used in conjunction with this article (freely available on the Web sites of PLoS Medicine at http://www.plosmedicine.org/, Annals of Internal Medicine at http://www.annals.org/, and Epidemiology at http://www.epidem.com/). Information on the STROBE Initiative is available at www.strobe-statement.org.
