# Supplementary material for: Perceived Stigma towards Leprosy among Community Members Living Close to Nonsomboon Leprosy Colony in Thailand
Source: PLoS One. 2015 Jun 5;10(6):e0129086. doi: 10.1371/journal.pone.0129086 (PMC4457619; doi:10.1371/journal.pone.0129086)
Supplement: S2 File — (DOCX) [file pone.0129086.s002.docx]

### Appendix S1: Leprosy research questionnaire for community members

**Interviewer ID: Study Code:**

**Part I:**

A. Socio-demographic characteristics

B. Knowledge and perceptions about leprosy

**Part II:**

Explanatory Model Interview Catalogue (EMIC-c)

- CASES:

**Inclusion criteria:**

- Male and female persons not affected by leprosy
- Age 18 or older
- Living in the community nearby for more than 1 year
- Able to communicate with the study investigator and to understand and answer the questions adequately
- Not willing to participate in the study

**Part I:**

A. Demographic entities:

1. Age: ………. years
2. Sex: □ female □ male
3. Ethnicity: □ Thai □ Other (………………..)
4. Village/Community: □ Non Somboon □ Other
5. How many years lived in the community? ………. years
6. Religion:
   1. □ Buddhism
   2. □ Christian
   3. □ Muslim
   4. □ Hindi
   5. □ other
7. Marital status:
   1. □ Unmarried
   2. □ Married
   3. □ Separated
   4. □ Widowed
8. Family medical history: family member diagnosed with leprosy?
   1. □ yes
   2. □ no
   3. □ don’t know
9. Anybody in close relationship, non-family, diagnosed with leprosy?
   1. □ yes
   2. □ no
   3. □ don’t know
10. School education:
    1. □ yes (literate)
    2. □ no (illiterate)

*If yes*, up to which level?

1. Primary level (< 5 years)
2. Secondary level (5-10 years)
3. Higher education (> 10 years)
4. Occupation
   1. □ farmer
   2. □ Laborer
   3. □ private business
   4. □ civil/office
   5. □ student
   6. □ house-wife/man
   7. □ unemployed
   8. □ other
5. Is your financial income enough to support your family sufficiently (total income)?
   1. □ yes
   2. □ no

B. Knowledge on leprosy:

1. Did you ever receive information on leprosy?
   1. □ Yes
   2. □ No

*If yes*, where did you get the information (several selections possible)?

1. □ hospital
2. □ health station
3. □ local doctor
4. □ TV/newspaper/radio
5. □ friend or family
6. □ other
7. Do you know what causes leprosy?
   1. □ yes
   2. □ no

*If yes,* please choose one of these:

- 1. □ Bacteria or any micro-organism
  2. □ Curse by god
  3. □ Karma
  4. □ Other: specify (_____________)

1. Do you think leprosy is very infectious?
   1. □ yes
   2. □ no
2. Do you know how leprosy is transmitted?
   1. □ yes
   2. □ no

*If yes*, how is it transmitted?

1. □ air
2. □ water soil
3. □ food
4. □ animal
5. □ mosquito
6. □ close contact to infectious persons
7. □ other
8. Do you think leprosy is difficult to treat?
   1. □ yes
   2. □ no
9. Do you know the signs and symptoms of leprosy?
   1. □ yes
   2. □ no

*If yes*, which of the following are signs and symptoms of leprosy (multiple answers possible!)?

- - 1. □ Pale or reddish patches on the skin
    2. □ Low or decreased sensitivity in these skin patches
    3. □ Weakness in hands feet or eyelids
    4. □ Pain in the nerves
    5. □ Swelling or lumps in the face or earlobes
    6. □ Painless wounds or burns on hands and feet

1. Do you think leprosy is a very severe disease?
   1. □ yes
   2. □ no
2. Do you think leprosy is a punishment (by God, etc.) for having done something bad in life?
   1. □ yes
   2. □ no

**Part II: “Explanatory Model Interview Catalogue” (EMIC-c) stigma scale for the community/non-affected, adjusted for leprosy.**

| **No.** | **Items** | **Yes** | **Possibly** | **Don’t know** | **No** | **Score** |
| --- | --- | --- | --- | --- | --- | --- |
|  |  | **3** | **2** | **1** | **0** |  |
| 1. | Would a person with leprosy keep others from knowing, if possible? |  |  |  |  |  |
| 2. | If a member of your family had leprosy, would you think less of yourself, because of this person’s problem? |  |  |  |  |  |
| 3. | In your community, does leprosy cause shame or embarrassment? |  |  |  |  |  |
| 4. | Would others think less of a person with leprosy? |  |  |  |  |  |
| 5. | Would knowing that someone has leprosy have an adverse effect on others? |  |  |  |  |  |
| 6. | Would other people in your community avoid a person affected by leprosy? |  |  |  |  |  |
| 7. | Would others refuse to visit the home of a person affected by leprosy? |  |  |  |  |  |
| 8. | Would people in your community think less of a family of a person with leprosy? |  |  |  |  |  |
| 9. | Would leprosy cause problems for the family? |  |  |  |  |  |
| 10. | Would a family have concerns about disclosure if one of their members had leprosy? |  |  |  |  |  |
| 11. | Would leprosy be a problem for a person to get married? |  |  |  |  |  |
| 12. | Would leprosy cause problems in an on-going marriage? |  |  |  |  |  |
| 13. | Would leprosy cause a problem for a relative of that person to get married? |  |  |  |  |  |
| 14. | Would leprosy cause difficulty for a person to find work? |  |  |  |  |  |
| 15. | Would people dislike buying food from a person affected by leprosy? |  |  |  |  |  |
